# Early detection of chronic renal disease: coordinated work between primary and specialized care in an ambulatory renal network of Peru

**DOI:** 10.1590/2175-8239-JBN-2018-0101

**Published:** 2019-03-07

**Authors:** Jessica Bravo-Zúñiga, Jungmei Gálvez-Inga, Pamela Carrillo-Onofre, Ricardo Chávez-Gómez, Paul Castro-Monteverde

**Affiliations:** 1 Edgardo Rebagliati Martins National Hospital Nephrologist Renal Health Unit Lima Peru Edgardo Rebagliati Martins National Hospital, Nephrologist Renal Health Unit, Lima, Peru.; 2 Juan José Rodríguez Lazo Polyclinic Lima Perú Juan José Rodríguez Lazo Polyclinic, Lima, Perú.

**Keywords:** Renal Insufficiency, Chronic, Mass Screening, Risk Factors

## Abstract

**Introduction:**

The aim of the study was to report the implementation of a functional network for the early diagnosis of chronic kidney disease (CKD) in patients with risk factors and the coordinated work between primary and specialized care in social security in Perú.

**Material and methods:**

A cross-sectional analysis of the data of patients evaluated in a health network in the city of Lima (2013 to 2016), older than 18 years, with risk factors for CKD, evaluated with serum creatinine and creatine albumin ratio in random urine (ACR). A multivariate logistic regression analysis was performed to evaluate the factors associated with the finding of CKD.

**Results:**

The implementation included training in renal health, installation of a digital database, organization of laboratories, and empowerment of primary care. We evaluated 42,746 patients of which 41.8% were men, with median age 69.2 years. The most frequent cause of detection was hypertension (HBP): 23,921 (55.9%). The prevalence of CKD was 12,132 (28.4%), the most frequent stage of CKD was 3a: 4735 (39.0%). Of the total, 6214 (14.5%) patients had microalbuminuria and 1335 (3.1%), macroalbuminuria. The risk of CKD increased 2.5 times (95% CI: 2.3-2.7) in patients with diabetes (DM) and HBP, in men (OR 1.2, 95% CI: 1.2-1.3) and as age increased (> 77 years: OR 2.7, 95% CI: 2.5-2.8). The identification of the disease in the primary care setting is 60% less likely than in specialized care.

**Conclusions:**

One of every four patients are diagnosed with CKD, and the simultaneous diagnosis of DM and HBP and old age are the most important factors.

## Introduction

Chronic kidney disease (CKD) is a public health problem. It is associated to a cardiovascular mortality risk of up to 8 to 10 times greater compared to the general population. Such risk increases with an associated reduction of the glomerular filtration rate (GFR)^1^. The risk of mortality in nephropathic patients increases as renal function decreases; when kidney function is lower than 30%, up to 46% of patients die and 54% develop stage 5 chronic kidney disease, with a close to null chance of survival without undergoing treatment^2^. As a result, a study of disease burden in our country placed CKD between the first 20 causes of healthy life years lost^3^.

In Peru, throughout the last decades, there has been a demographic and epidemiologic transition of the general population due to the increase of life expectancy and the greater prevalence of cardiovascular risk factors (hyperglycemia, high blood pressure (HBP), obesity and dyslipidemia), leading to a greater risk of the elderly to suffer from CKD^4^. Thus, when analyzing the data regarding the risk factors for CKD in Peru, the prevalence of HBP extends from 10 to 27.3%, depending on whether it is an urban or rural population; concurrently, the prevalence of diabetes mellitus (DM) ranges from 2.8 to 8%^5^. The latest study from Francis et al. conducted in Lima in 2015^6^ reports higher risk factor prevalence in the population over 35 years old: 9.9% for diabetes and 29.2% for HBP.

Furthermore, in recent years, the number of patients who enter renal replacement therapy or arrive with late stages of kidney disease to nephrology services has increased. In a narrative review of CKD in Peru, Herrera et al. outline that the diagnosis and management of this condition is deficient^7^: only 8.9% of the patients with DM are tested for albuminuria, about half of the patients on hemodialysis are informed of their diagnosis at the time of their admission to dialysis, and only 24% have had at least two evaluations of a nephrologist in the previous year.

As CKD frequently develops asymptomatically, adequate assessment of renal function is essential. Early identification of patients with kidney failure allows prompt treatment to stop the progression of kidney damage, managing to modify the associated risk factors that contribute to raise the morbidity in these patients^8^.

Although timely intervention of specialists is needed to improve the health of patients with CKD, it is also essential to avoid unnecessary referrals that could deplete the resources of the healthcare system^9^. Therefore, primary healthcare professionals should be trained to efficiently diagnose the condition and make a timely and adequate referral. A systematic review of the evidence on cost-effectiveness in early vs. late referral to the nephrologist found that early referral is associated with better health outcomes and could be more cost-effective^10^.

Therefore, a model of care with a structured program is required where both the primary healthcare physician and the specialist work together in the detection and management of CKD. In such model, the nephrologist intervenes in the direct management of patients with advanced stages of the disease and in the training of primary healthcare personnel for the supportive care of the renal patient. Simultaneously, the primary healthcare physician participates in the screening and management of patients with early stages of kidney disease.

This study reports the results from the largest healthcare network in Peru, the Rebagliati health network located in Lima, which serves 23% of the population that has social security in Peru (30% of the Peruvian population). It provides coverage to people with stable work and to retired people; generally the patients who are cared for are old and with multiple pathologies.

**Figure 1:**
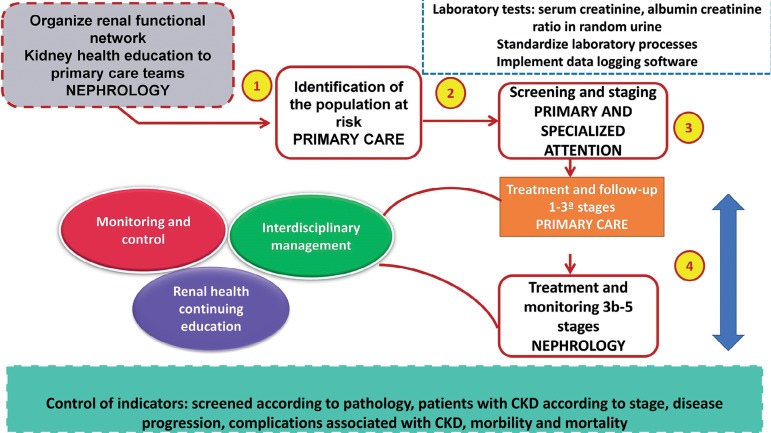
Implementation strategy of an early detection model for CKD in the Rebagliati Health Network.

A functional renal network was implemented, which included 14 primary care centers and two hospitals that are specialized in renal care. One of them is a hospital of high complexity and a national reference. A multidisciplinary renal healthcare team was established in each hospital (doctor, nurse, nutritionist, and psychologist). The healthcare network was subdivided into 4 territorial micro-networks, which were led by two nephrologists responsible for the on-site training of personnel in the diagnosis and management of CKD, as well as the registration of patients and in the referral and cross-referral criteria between healthcare facilities. Four laboratories were designated for the standardized processing of screening tests: creatinine blood test and the random albumin-creatinine ratio (ACR). Likewise, each center in the network had a software for the registration and follow-up of the patients evaluated. The management of diagnosed patients, after the selection was supervised by primary care for stages 1 to 3a and specialized care for stages 3b to 5. After each evaluation, a digital record was made. We have summarized the implementation strategy of an early detection model of CKD in Figure 1.

The study was reviewed and approved by the Ethics Committee of the Hospital Nacional Edgardo Rebagliati Martins, Lima Peru.

## Materials and methods

A retrospective secondary analysis of the database of electronic medical records of patients treated in outpatient renal health services of the Rebagliati health network between January 1, 2013 and November 30, 2016 was performed. The data included patients over 18 years of age, of both sexes, with risk factors for CKD: DM and / or HBP and / or over 55 years of age, who had laboratory results of the blood creatinine test and ACR as part of the evaluation of the ERC.

During the study period, patients were evaluated with a peripheral blood sample after an 8-hour fast. Serum creatinine concentrations were determined by the compensated Jaffe creatinine method (Roche Diagnostics) that has calibration traceable to an IDMS reference measurement procedure. Creatinine values were expressed in mg/dL. All the patients left their first morning urine in the different healthcare centers and the samples were sent to the designated laboratories. After centrifuging, the samples were processed in an automated modular biochemistry analyzer (P 800, Hitachi) with albumin and creatinine reagents (Roche Diagnostics) for the quantitative determination of these analytes in urine. The results obtained were validated and entered into the database.

### Variable definition

Chronic kidney disease (CKD): according to the definition of the National Kidney Foundation - Kidney Disease Outcomes Quality Initiative (US NKF-KDOQI), CKD is diagnosed when the patient meets any of the following criteria: decreased renal function expressed by a GFR < 60 mL/min/1.73 m^2^ and/or the presence of a renal impairment marker (ACR>30 mg/g), independently of the underlying cause of CKD, for a duration longer than 3 months. The estimated glomerular filtration rate (eGFR) was calculated with the equation MDRD-4 from the Modification of Diet in Renal Disease study: eGFR = 186 × Creatinine - 1.154 × (Age) - 0.203 × (0.742 if the patient is female) (mL/min/1.73m^2^)^11^.

Renal impairment marker (ACR): Results from the division of albumin by creatinine measured in urine expressed in mg/g was considered as a marker for this study. According to the Kidney Disease: Improving Global Outcomes (KDIGO) 2012 clinical practice guideline, kidney damage can be classified as follows: A1: ACR < 30 mg/g (normal or slightly increased), A2: ACR between 30-300 mg/g (moderately increased), A3: ACR > 300 mg/g (severely increased). The combination of both diagnostic criteria allows classifying CKD in 5 stages from lower to higher functional compromise^12^.

We defined a patient with DM as one who previously had this diagnosis, has a glycosylated hemoglobin (HbA1c) level of 6.5% or higher, or with a fasting plasma glucose > 126 mg/dL or a random plasma glucose test > 200 mg/dL, or those who were taking antidiabetic medications. A patient with HBP was defined as the one who previously had this diagnosis, has a blood pressure > 140/90 mmHg, and those who were taking antihypertensive medication.

## Statistical analysis

The Mann Whitney U test was applied to compare continuous variables and the chi-square test was used for categorical variables. The continuous variables are presented as median and interquartile range (IQR) since they had skewed distribution. The categorical variables are presented as frequencies and percentages. The odds ratio (OR) and the 95% confidence interval (95%CI) were calculated with multivariate models to quantify the association between chronic kidney disease and potential predictor variables (age, etiology of disease, sex, and healthcare setting at the time of diagnosis). The full model included variables that had a *p* value < 0.20 in the bivariate analysis (standard value by convention) and the ones that were considered biologically relevant. A value of *p* < 0.05 was considered statistically significant. The statistical analyzes were performed using STATA version 13 (STATA Corp., TX, USA).

## RESULTS

### Population characteristics and CKD stages

In the study period, 50,285 patients were evaluated; 12 patients were excluded from the analysis because they were under 18 years old and 7,527 patients because their complete data was not available. The final sample included 42,746 patients.

The demographic, clinical, and laboratory parameters of the population are detailed in Table 1: 32,259 (75.5%) patients were screened in primary care. The population was mostly female (24,883, 58.2%), the median age was 69.2 years (IQR 59.9-77.2), and 25,762 (60.3%) patients were pensioners. Hypertensive patients (23,921, 55.9%) were the ones who performed CKD screening tests most frequently. Considering the two laboratory parameters for the diagnosis of CKD (ACR and eGFR), 12,132 (28.4%) patients met the diagnostic criteria; the frequency of CKD in patients with HBP was 55.9% and in patients with DM plus HBP was 20%. Prevalence increased progressively with age, from 17% in the group under 60 years old to 36.6% in those older than 77 years.

**Table 1:** Demographic, clinical, and laboratory characteristics of the participants according to the finding of chronic kidney disease (CKD), in the Rebagliati Martins Health Network, 2013-2016

Characteristics	Total n=42 746	No CKD n= 30 614(71.6%)	CKD n=12 132(28.4%)	*p*
Age (years)	69.2 (59.9-77.2)	73.2 (64.5-80.3)	67.7 (58.5-778)	< 0.001*
< 60	10,739 (25.1)	8,675 (28.3)	2 064 (17)	< 0.001*
60-69	10,355 (24.2)	7,974 (26)	2 381 (19.6)	< 0.001*
69-77	10,594 (24.8)	7,349 (24)	3 245 (26.7)	< 0.001*
> 77	11,058 (25.9)	6,616 (21.6)	4 442 (36.6)	< 0.001*
Sex				
Male	17,863 (41.8)	12 219 (39.9)	5 644 (46.5)	< 0.001*
Female	24,883 (58.2)	18 395 (60.1)	6 488 (53.5)	< 0.001*
Etiology				
Only Diabetes *Mellitus*	6,342 (14.8)	4,641 (15.2)	1,701 (14.0)	0.003*
Only Hypertension	23,921 (55.9)	17,136 (56)	6,785 (55.9)	0.928*
Diabetes *Mellitus* + Hypertension	6,131(14.3)	3,708(12.1)	2,423 (20)	< 0.001*
Other diagnoses	6,352 (14.9)	5,129 (16.8)	1,223 (10.1)	< 0.001*
Retired insurance	25,762 (60.3)	17,712 (57.8)	8,050 (66.3)	< 0.001*
Type of Healthcare				
Primary Care	32,259 (75.5)	24,160 (78.9)	8,099 (66.7)	< 0.001*
Specialized Care	10,487 (24.5)	6,454 (21.1)	4,033 (33.3)	< 0.001*
Laboratory tests (RIC)				
Seric Creatinine	0.8 [0,7-1.0]	0.8 [0.7-0.9]	1.2 [0.9-1.4]	< 0.001^†^
RAC	6.2 [7-17.8]	4.7 [2.2-9.9]	41.4 [9.6-112.6]	< 0.001^†^
TFG e	81.6 [67-97.7]	86.2 [74.6-100.9]	58.3 [49.6-82.1]	< 0.001^†^

Data are reported as the mean [Interquartile Range] or the number of patients (percentage). CKD: chronic kidney disease; RAC: albumin creatinine ratio in urine; eGFR: estimated glomerular filtration rate.

† Rank sum test,

* chi-square test.

The CKD stage most frequently found in patients at the time of diagnosis was 3a: 4,735 (39%). When evaluating the ACR laboratory results, 6,214 (14.5%) patients presented moderate increase (A2) and 1,335 (3.1%) severe increase (A3). When the GFR and ACR results were interpreted to establish CKD staging and prognosis, 1,478 (12.2%) patients had a higher risk of progression; these patients included those with advanced CKD and those with high levels of albuminuria (Table 2).

**Table 2:** Staging of chronic kidney disease and albuminuria level, in the Rebagliati Martins Health Network, 2013-2016

CKD STAGE	Albuminuria category (albumin/creatinine index in urine (mg/g))			
	A1 NORMAL < 30 mg/g	A2 MODERATE INCREASE 30-300 mg/g	A3 SEVERE INCREASE > 300 mg/g	Total patients according to stage
Stage 1	13060 (37.1)	1968 (31.6)	271 (20.3)	2239 (18.5)
Stage 2	17554 (49.9)	2629 (42.3)	436 (32.7)	3065 (25.3)
Stage 3a	3531 (10)	968 (15.6)	236 (17.7)	4735 (39.0)
Stage 3b	851 (2.4)	463 (7.5)	207 (15.5)	1521 (12.5)
Stage 4	162 (0.5)	145 (2.3)	129 (9.7)	436 (3.6)
Stage 5	39 (0.1)	41 (0.7)	56 (4.2)	136 (1.1)
	35197 (82.3)	6214(14.5)	1335(3.1)	12132

Patients without CKD 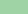
Mild risk of progression of CKD 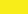
Moderate risk of progression of CKD 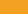
Severe risk of progression of CKD 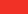
A1: albuminuria < 30 mg/g, A2: albuminuria > 30 mg/g and < 300 mg/g, A3: albuminuria ≥ 300 mg/g Stage 1: TFG > 90 mL/min and albuminuria >30 mg/g, A2 or A3 Stage 2: TFG > 60 and < 90 mL/min and albuminuria > 30 mg/g, A2 or A3 Stage 3ª: TFG > 45 mL/min and < 60 mL/min with or without albuminuria > 30 mg/g Stage 3b: TFG < 45 mL/min and > 30 mL/min with or without albuminuria > 30 mg/gw Stage 4: TFG < 30 mL/min with or without albuminuria > 30 mg/g Stage 5: TFG < 15 mL/min with or without albuminuria < 30 mg/g (11)

### Associated factors with CKD findings

A positive correlation was observed between CKD and male population (OR 1.2, 95%CI: 1.2-1.3); the risk raised as the age increased: approaching a risk of 2.7 times (95%CI: 2.5-2.8) in patients older than 77 years. Likewise, having diabetes and hypertension increased the risk of CKD (OR 2.5, 95%CI: 2.3-2.7) while there was a 60% lower probability of finding patients with CKD in primary care than in specialized care (OR 0.4, 95%CI: 0.2-0.6) (Table 3).

**Table 3:** Risk factors for chronic kidney disease in screening in the Rebagliati Martins Health Network, 2013-2016

Characteristics	CRUDE OR (95%CI)	ADJUSTED OR * (95%CI)
Sex		
Male	1.3 (1.2-1.4)	1.2 (1.2-1.3)
Female	1	1
Age		
< 60	1	1
60-69	1.2 (1.2-1.3)	1.2 (1.1-1.3)
70-77	1.8 (1.7-2.0)	1.7 (1.6-1.9)
> 77	2.8 (2.6-3.0)	2.7 (2.5-2.8)
Etiology		
Only Diabetes *Mellitus*	1.4 (1.3-1.4)	1.7 (1.6-1.9)
Only Hypertension	1.4 (1.3-1.4)	1.4 (1.3-1.5)
Diabetes Mellitus + Hypertension	1.8 (1.7-1.9)	2.5 (2.3-2.7)
Other diagnoses	1	1
Type of Healthcare		
Primary Healthcare (primary care)	0.5 (0.2-0.6)	0.4 (0.2-0.6)
Secondary Healthcare (medical specialists)	1	1

OR: Odds Ratio, CI: confidence interval, CKD: Chronic kidney disease

* Model adjusted by sex, age, type of healthcare, and hypertension. Variables that had a *p* < 0.20 in the crude model and were considered biologically relevant were included.

## Discussion

Our study describes the results of the implementation of a CKD detection program based on 4 fundamental pillars: constant training in renal health topics for primary care professionals led by nephrologists, installation of a database that helps in the search and follow-up of patients, implementation of laboratories for sampling, and above all, cooperative work between primary and specialized care. Our results confirm that advanced age and comorbidities such as diabetes and hypertension are factors that increase the risk of CKD.

In Latin America, few countries have national programs to prevent CKD. According to Cusumano's report in 2008, only Brazil, Cuba, Uruguay, Venezuela, and Peru have initiated such programs^13^. Since then, the progress has been different: in Colombia, the design and implementation of the prevention program has been based on the integration of service networks and levels of care^14^, in such a way that 74.9% of patients insured by the healthcare system who have HBD and/or DM had already been evaluated for CKD in 2013^15^. In Uruguay, the program was planned and designed by the Uruguayan Society of Nephrology, following the recommendations of the Latin American Society of Nephrology and Hypertension (SLANH), which includes a shared work between primary and specialized care^16^. In Cuba, CKD and other chronic noncommunicable diseases that lead to systemic vascular damage are the subject of priority attention, ranging from the execution of intersectoral and multidisciplinary operations to the control and surveillance of risk factors^17^. However, in Peru, the renal health program was only partially developed in the social security healthcare system through the National Renal Health Plan approved in 2008, whose objectives were: decrease the incidence of CKD in the population at risk, organize health care, strengthen the resolution capacity of primary care, and establish a surveillance system for renal health^18^.

The prevalence of CKD in the general population varies according to the geographical area between 8 and 13%^19^. However, if we consider risk groups, as the population of our study, prevalence increases. Most studies report that the prevalence of CKD in diabetic patients varies between 37 and 40% when we simultaneously evaluate the presence of proteinuria and/or the fall of the GFR below 60 mL/min/1.73m^2^^20^.

This was also reported in a study conducted in the primary care setting of the social security healthcare system in Peru, concluding that 24.4% of diabetic patients and 20.2% of hypertensive patients evaluated with GFR (calculated by MDRD4) and 24-hour urine protein test presented CKD^21^.

Regarding CKD in hypertension, a study conducted by primary care physicians in Spain reports that 53.9% of patients with GFR< 60 mL/min have HBP^22^. In Colombia, Acuña describes that 48.1% of hypertensive patients have CKD^15^. We found a prevalence of CKD of 26.8% in diabetic patients, 28.4% in hypertensive patients, and 39.5% in patients with both conditions.

The median age of our patients with CKD was 73 years. In the NHANES report, the prevalence of CKD in patients older than 70 years with a GFR below 60 mL/min/1.73m^2^ was 37.8% between 1999 and 2004^23^. In a study conducted in the United States in a population older than 65 years, the prevalence of GFR < 60 was 22%^24^. In our study, the GFR in patients older than 60 years was 23% and it raised progressively as age increased so the prevalence in those over 77 years reached 40%. Age is one of the most important factors that affect kidney function. The GFR decreases by 1 mL/min/1.73m^2^ per year after the age of 30 years in healthy people^25^. The decrease of the GFR could be due to changes in renal structure associated with aging^26^.

The percentage of the different kidney damage classifications established by ACR results is similar in all the studies reviewed. In a Korean study^27^, which evaluated healthy adults, it was found that 93.5% of the patients were A1 (ACR < 30 mg/g), 5.6% A2 (ACR 30-300 mg/g) and 0.9% A3 (> 300 mg/g). The Peruvian Society of Nephrology in 2012 conducted a descriptive study on the frequency of albuminuria in patients with known risk factors, finding a prevalence of 53.4% of microalbuminuria, using urine test strips as the diagnostic procedure, a qualitative method that may increase false positive results^28^.

Figueroa et al. evaluated microalbuminuria with ACR results in patients older than 55 years with risk factors from the social security healthcare system in Peru, detecting A2 in 17.9% and A3 in 5.4%^29^. We found that 14.5% of patients had A2 and 3.1% A3. High albuminuria levels are a sign of kidney injury and, together with the GFR value, establishes the ground on which the diagnosis of CKD is based; both are indicators of CKD progression^30^.

When evaluating the different stages of CKD in our population, we found that stages 1 to 3A (GFR > 45 mL/min) constituted 82.6% of cases, which should be evaluated and followed-up in primary care centers. However, when evaluating each stage independently, the most frequent was 3A in 39%, a result that matches the results published by Benghanem et al. in Morocco who found that 3A is the most prevalent CKD stage of its study population: 40.2%^31^, and with Acuña's study who reports that in the CKD record of Colombia, 94.3% of patients are in stages 1 to 3, with 43.8% in stage 3^15^.

An important aspect to consider in CKD is that a GFR below 60 mL/min/1.73m^2^ and an ACR > 30 mg/g independently predict the mortality risk and cardiovascular events that can be explained by endothelial dysfunction, bone mineral alteration, inflammation, and anemia^32^.

The protective factor of identifying the disease at the first level of care makes early intervention necessary, reinforcing the need for a shared work between primary and specialized care and the creation of renal health networks that ensure patient care at any stage of sickness.

As Cusumano states^33^, a CKD prevention program requires the implementation of public health strategies, to not only diagnose and treat, but also educate the population and perform screening at early stages, thus, lowering disease burden. As suggested by Buch, a multifactorial approach is required with the application of all possible preventive measures for CKD^17^.

A restriction to achieve this is the lack of public policies that encourage healthy lifestyles in the general population and that now medical practice programs are mainly focused on treatment, with emphasis on dialysis (with non-universal coverages), dismissing primary and secondary prevention programs. There are also structural and organizational limitations of the healthcare system for the general and vulnerable populations, and it has been demonstrated that access barriers to healthcare services interfere with quality care and limit the clinical protection of patients, increasing social and health inequity^34^. In the case of the Peruvian healthcare system, inequality is evident because there are different healthcare systems that end up establishing a gap between people from a lower and upper socioeconomic level. For CKD, financial barriers decrease access to healthcare services; in addition, those who access the healthcare service do not always continue or complete their treatment.

The main strength of our work is its representativeness because it has been carried out in the largest healthcare network in the country, which has allowed identifying patients with a higher risk of kidney disease. The results contribute with the design of strategies focused on this subgroup, especially those based on the shared work of primary and specialized care, achieving the empowerment of primary care physicians in renal health topics and allowing the efficient use of specialized services.

This study also has some limitations. The underreporting of data might have occurred, because some professionals were not yet aware of the digital filling of clinical information. Moreover, inadequate referral to the nephrologist and loss of patients during follow-up were considered study limitations.

## Conclusions

CKD in at-risk population affects 1 in every 4 patients evaluated. It is more commonly found in early stages, and age and simultaneously having DM and hypertension increase the risk. The diagnosis of CKD in primary care contributes with its early identification.
